# Nanoparticles Obtained from Zein for Encapsulation of Mesalazine

**DOI:** 10.3390/pharmaceutics14122830

**Published:** 2022-12-16

**Authors:** Izabela Borges C. Lima, Lina Clara G. A. I. Moreno, Ana Victória Peres, Ana Cristina Gramoza Santana, Adonias Carvalho, Mariana H. Chaves, Lorena Lima, Rayran Walter Sousa, Dalton Dittz, Hercília M. L. Rolim, Lívio César Cunha Nunes

**Affiliations:** 1 Laboratory of Technological Innovation, Entrepreneurship, Medicines and Related (LITE), Nucleus of Pharmaceutical Technology, Federal University of Piauí, Teresina 64049-550, PI, Brazil; 2 Pharmaceutical Nanosystems Laboratory (NANOSFAR), Nucleus of Pharmaceutical Technology, Federal University of Piauí, Teresina 64049-550, PI, Brazil; 3 Natural Products Laboratory (LPN), Department of Chemistry, Federal University of Piauí, Teresina 64049-550, PI, Brazil; 4 Laboratory of Experimental Cancerology (LabCâncer), Nucleus of Pharmaceutical Technology, Federal University of Piauí, Teresina 64049-550, PI, Brazil

**Keywords:** natural polymer, zein, 5-ASA, nanoparticles, colon

## Abstract

We encapsulated MSZ in zein nanoparticles (NP-ZN) using a desolvation method followed by drying in a mini spray dryer. These nanoparticles exhibited a size of 266.6 ± 52 nm, IPD of 0.14 ± 1.1 and zeta potential of −36.4 ± 1.5 mV, suggesting colloidal stability. Quantification using HPLC showed a drug-loaded of 43.8 µg/mg. SEM demonstrated a spherical morphology with a size variation from 220 to 400 nm. A FTIR analysis did not show drug spectra in the NPs in relation to the physical mixture, which suggests drug encapsulation without changing its chemical structure. A TGA analysis showed thermal stability up to 300 °C. In vitro release studies demonstrated gastroresistance and a sustained drug release at pH 7.4 (97.67 ± 0.32%) in 120 h. The kinetic model used for the release of MSZ from the NP-ZN in a pH 1.2 medium was the Fickian diffusion, in a pH 6.8 medium it was the Peppas–Sahlin model with the polymeric relaxation mechanism and in a pH 7.4 medium it was the Korsmeyer–Peppas model with the Fickian release mechanism, or “Case I”. An in vitro cytotoxicity study in the CT26.WT cell line showed no basal cytotoxicity up to 500 μg/mL. The NP-ZN showed to be a promising vector for the sustained release of MSZ in the colon by oral route.

## 1. Introduction

**Figure 1:**
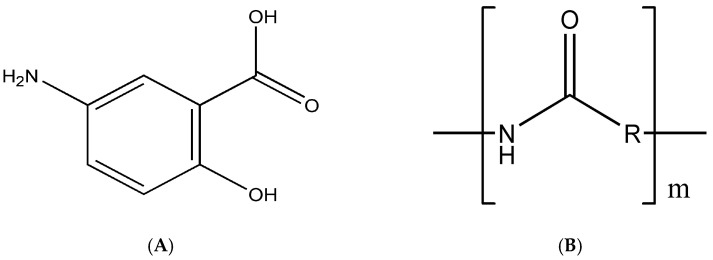
Chemical structure of mesalazine (**A**) and zein (**B**).

Mesalazine (MSZ), also known as mesalamine or 5-aminosalicylic acid (5-ASA) (Figure 1A), is an aminosalicylate widely used in IBD to induce remission and maintenance, especially in mild to moderate ulcerative colitis. Its mechanism of action is not fully understood, but it involves the suppression of NF-kB, protection of the epithelial barrier of T84 cells against peroxynitrites, reduction in free radicals and inhibition of leukotrienes, prostaglandins and proinflammatory cytokines [1,2].

In addition to its high cost, this drug has the following limiting factors to its use: the dosage regimen reaches 4.8 g/day, low absorptivity in the inflamed colon and greater systemic absorption, which favors adverse effects and makes the patient’s adherence to the treatment difficult [3,4].

The size of the formulation is inversely proportional to the specificity for the inflamed tissue [5]. Polymeric nanoparticles (NPPs) offer a greater selectivity of the drug to the target organ, resulting in higher, more effective and longer-lasting concentrations compared to conventional delivery systems. In addition, these nanoparticles protect against degradation, and possess a high encapsulation capacity and greater stability when in contact with biological fluids [6,7].

Zein is a natural protein polymer, the main storage protein of corn seeds (*Zea mays* L.), classified as prolamin (Figure 1B); it is rich in nonpolar amino acids (e.g., leucine, alanine and proline) and also contains some polar amino acid residues (e.g., glutamine), resulting in its high solubility in aqueous ethanol solutions (90%) and low solubility in water only [8,9].

Due to being obtained naturally, it has advantages such as biodegradability, biocompatibility and ability to develop mucoadhesive interactions, which remain in contact with the absorptive surface of enterocytes for a longer time, promoting the controlled release of drugs [10]. In addition, this natural polymer has been approved by the Food and Drug Administration (FDA) as one of the safest biomaterials [11].

Due to its unique amphiphilic character, zein can easily self-assemble to form nanoparticles for the release of hydrophobic bioactive ingredients. Zein NPs can significantly improve drug solubility and stability, as well as extend drug action time, promoting targeted drug delivery, improving efficacy and reducing toxicity and adverse reactions [12,13].

Its nanoparticles have already been studied to carry several drugs: quercetin [10,14], paclitaxel [15], beta carotene [16], eugenol [17], terpinen-4-ol [18], α-tocopherol [19], 5-fluorouracil [11], cannabidiol [20], vitamin D3 [21], curcumin [8,22], lutein [23], mint oil [24], thymol [25], daidzin [26], pterostilbene [9], among others. The present work aims to develop and characterize nanoparticles from zein for the oral delivery of mesalazine.

## 2. Materials and Methods

### 2.1. Materials

Zein, lysine and mannitol were purchased from Sigma-Aldrich (St. Louis, MO, USA). Mesalazine was purchased from Henri-farma, Teresina, Brazil, lot number 10100025. Ethanol was obtained from Merck (Darmstadt, Germany). All reagents and chemicals used were of analytical grade.

### 2.2. Preparation of Zein-Nanoparticle-Loaded Mesalazine (NP-ZN-MSZ)

**Figure 2:**
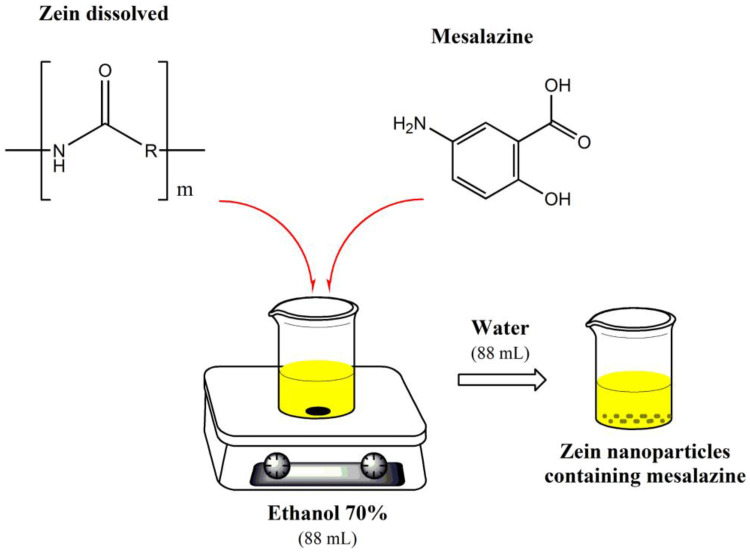
Schematic representation of the preparation of NP-ZN-MSZ.

Zein nanoparticles (NP-ZN) were prepared using a desolvation method with subsequent spray drying [10]. Initially, 600 mg of zein and 60 mg of lysine were dissolved in 63 mL of a 70% ethanol solution. Lysine was used to improve the colloidal stability and redispersibility [27]. In parallel, 30 mg of mesalazine (MSZ) was solubilized in 25 mL of a 70% ethanol solution (totaling 88 mL of the 70% ethanol solution) under stirring at 37 °C for 30 min and added to the lysine–zein solution under magnetic stirring. After 30 min of incubation, nanoparticles were formed by adding 88 mL of purified water. Then, 12 mL of a 10% aqueous mannitol solution was added. The summarized representation is in Figure 2.

The nanoparticle suspension was dried in a spray dryer (Buchi Mini Spray Dryer B-290, Buchi Labortechnik AG, Flawil, Switzerland) under the following experimental conditions: (i) inlet temperature, 90 °C; (ii) outlet temperature, 45–50 °C; (iii) air pressure, 2–5 bar; (iv) pumping rate, 5 mL/min; (v) aspirator, 100%; and (vi) air flow, 900 L/h.

### 2.3. Size, Particle Distribution and Polydispersion Index

The mean diameter and particle distribution were determined using dynamic light scattering (DLS) using a laser particle counter calibrated with standard particle size latex suspensions. The sample was diluted with ultrapure water and the reading was performed at room temperature (20 ± 1 °C) and at a fixed angle of 90° using a Zeta-Sizer SZ90 (Malvern^®^ Instruments, Malvern, UK).

### 2.4. Zeta Potential

To determine the surface charge of the nanoparticles, the samples were diluted (1:10) in ultrapure water and analyzed using laser Doppler anemometry on a Zetamaster SZ90 electrophoretic scattering analyzer (Malvern^®^ Instruments, Malvern, UK). The zeta potential was expressed in millivolts (mV).

### 2.5. Dosing of the Drug in the Nanoparticles

For the dosage of the encapsulated drug, the NPs were centrifuged at 5000 rpm for 20 min, forming a supernatant (free drug) and a precipitate (encapsulated drug). The supernatant was quantified using HPLC. For a total quantification of the drug in the formulation, the NPs were lysed with ethanol PA, making them clear, and then quantified using HPLC.

### 2.6. Quantification of Mesalazine using HPLC

The quantification of mesalazine was performed according to [28] using HPLC (Shimadzu A20, Kyoto, Japan). A C18 column (4.6 × 250 mm, 5 μm Shim-pack particle) was used for chromatographic separation and drug determination. Ultrapure water (pH 5.5 modified with 0.1 M citrate buffer) and acetonitrile (HPLC grade) 85:15 (*v*/*v*) indicated the mobile phase that was previously filtered, at a flow of 1 mL/min, fraction of injection 20 µL and working pressure of 200 gf. The UV/Vis detector was set at 300 nm. The mesalazine showed a retention time of 3.4 min. The standard curve was used to quantify mesalazine in a different medium, according to the following equations:

y = 15,067x + 48,855 (R^2^ = 0.9986) in HCl 0.1M pH 1.2 (1)

y = 6174.5x + 10,430 (R^2^ = 0.992) in buffer pH 6.8 (2)

y = 13,657x − 49,653 (R^2^ = 0.9965) in buffer pH 7.4 (3)

where x is the MSZ concentration (μg/mL) and y is the peak area. A linear correlation was observed in the concentration range between 4 and 256 μg/mL. Mesalazine standard solutions (400 µg/mL) in different media (HCl 0.1M pH 1.2), a buffer solution of pH 6.8 and a buffer solution of pH 7.4 [29], were used for the standard curve.

### 2.7. Encapsulation Efficiency (EE%)

To calculate the EE%, the following equations were used:

Q_total_ = Q_supernatant_ + Q_encapsulated_ (4)

EE (%) = (Q_encapsulated_/Q_total_) × 100 (5)

Q_total_ is the total amount of drug incorporated into the formulation;

Q_encapsulated_ is the amount of drug encapsulated in the nanoparticles;

Q_supernatant_ is the amount of free drug that was not encapsulated in the nanoparticles.

### 2.8. Scanning Electron Microscopy (SEM)

Scanning electron microscopy (SEM) was performed to determine the morphology and characteristics of the polymeric surface using the FEI field emission electron source equipment (SEM-EC), model FEG-250. Polymers are sensitive to the electron beam, so the samples were previously metallized with aluminum [30]. The NPs were placed in a stub wrapped with aluminum foil and metallized with gold. The equipment was configured for 15 KV and spot 3.

### 2.9. Fourier Transform Infrared Spectroscopy (FTIR)

The Fourier transform infrared spectroscopy (FTIR) of the powdered nanoparticles was obtained using the Perkin Elmer Spectrum 100 GTX spectrophotometer on KBr pellets. The analysis was performed in the 4000 to 400 cm^−1^ region with a resolution of 4 cm ^−1^ and 16 scans in transmittance mode.

### 2.10. Thermogravimetric Analysis (TGA)

The thermogravimetric analysis was performed with the SDT Q600 equipment from TA Instruments. For each test, 6.00 ± 1.00 mg of the sample was heated at room temperature to 700 °C at a heating rate of 10 °C/min, under an argon atmosphere, with a flow rate of 50.00 mL/min and aluminum-treated samples.

### 2.11. In Vitro Release Kinetics Study

The drug release profile from zein nanoparticles (NP-ZN-MSZ) was evaluated using the dynamic dialysis method [31]. Briefly, cassette^®^-type cellulose acetate dialysis membranes were used under sink conditions, where the drug concentration was <20% of its solubility in a 0.1M HCl solution pH 1.2 (>18 mg/mL), phosphate-buffered solution pH 6.8 (~3.5 mg/mL) and phosphate-buffered solution pH 7.4 (~5.5 mg/mL) at 37 °C to avoid the saturation of the medium [32].

The NP-ZN-MSZ sample (1.5 mL at 262µg/mL of the drug) was introduced into the cellulose acetate membrane with 10,000 Da molecular exclusion pores and dialyzed against 250 mL of gastric medium (pH 1.2), intestinal (pH 6.8) and simulated the ileocolonic region (pH 7.4) [29] at 37 °C in orbital agitation for 120 h. An amount of 1.5 mL of aliquots was removed from the medium at set times and the MSZ concentration was quantified using HPLC, as described above. The buffer was replenished to keep the volume constant.

Quantifications at 300 nm were converted into the percentage of drug released according to the standard curve of each previously established medium for which linearity was confirmed. The experiment was performed in triplicate and drug concentrations were corrected considering the dilution factor.

The parameters to determine the release kinetics of the nanoparticles were statistically analyzed using the Excel DDSolver supplement. The model was selected according to the adjusted correlation coefficient (R^2^adj).

### 2.12. In Vitro Cytotoxicity Study

The MTT ([3-(4,5-dimethylthiazol-2yl)-2,5-diphenyl tetrazolium] bromide) assay proceeded as described by [33]. Initially, the cell line used (CT26.WT, murine colon carcinoma) was incubated with the usual varying concentrations of NP-ZN, and NP-ZN-MSZ was tested at a density of 5 × 10^3^ cells/well in 96-well plates for 72 h at 37 °C and 5% CO_2_. The positive control was performed with doxorubicin (10 µM). After the incubation period, 20 mL of the MTT solution (5 mg/mL) was added to the cultures and the plates were reincubated for 4 h in an oven at 37 °C and 5% CO_2_. After this period, the supernatant was discarded with the culture medium and the precipitate was resuspended with 100 mL DMSO for 5 min under agitation. Then, the plates were read in an ELISA spectrophotometer at 560 nm.

### 2.13. Statistical Analysis

Statistical analyses were performed using ANOVA and Tukey’s test. All data were obtained from tests in triplicate and expressed as mean ± standard deviation. Statistical significance for this study was considered when *p* < 0.05.

## 3. Results and Discussion

### 3.1. NP Preparation and Characterization

**Figure 3:**
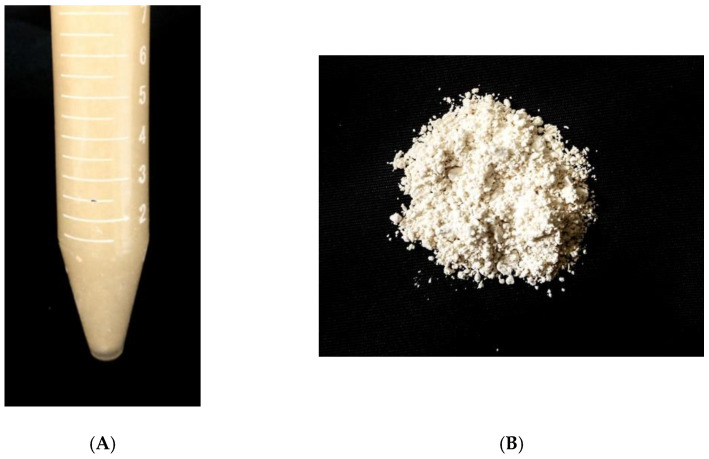
Nanoparticle formation (**A**) and after drying in spray dryer (**B**).

Initially, the polymer–drug ratios (1:10; 1:12, 1:15 and 1:20) were tested; however, there was precipitation due to the saturation of the drug in relation to the polymer, with no formation of nanoparticles. Therefore, the best drug–polymer ratio was 1:20 (30 mg of drug and 600 mg of zein). The formation of nanoparticles was confirmed by the presence of the Tyndall effect [31], characterized by the visualization of an opalescent suspension with a milky appearance (Figure 3A).

**Figure 4:**
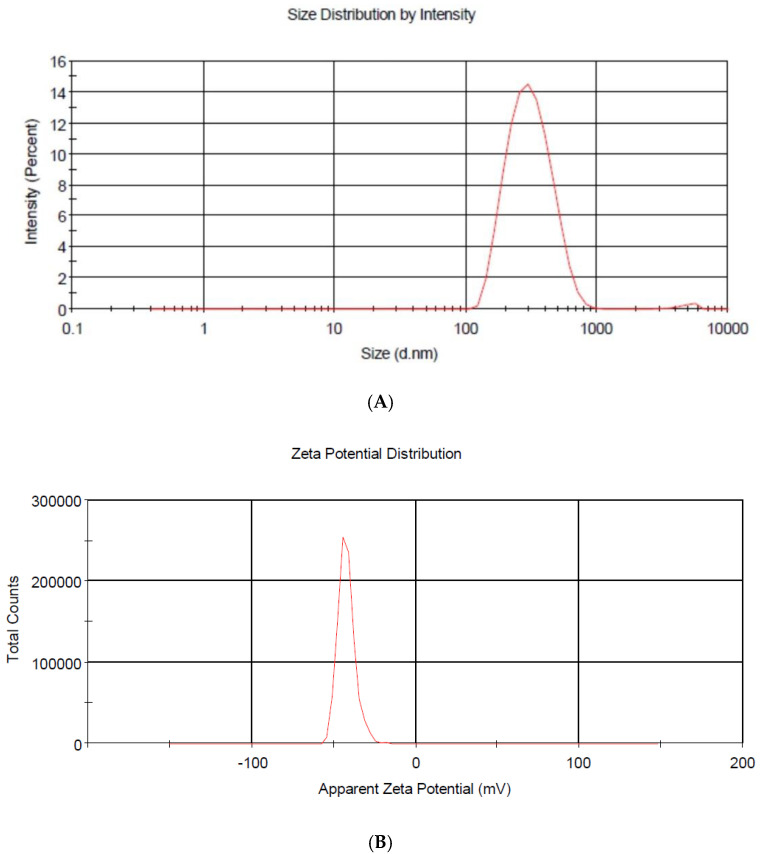
Particle size distribution (**A**) and zeta potential of NP-ZN-MSZ (**B**).

Through the dynamic light scattering method (DLS), the NP-ZN-MSZ showed a size of 266.6 ± 52 nm, and the empty nanoparticle (NP-ZN) was of a smaller size (218 ± 23 nm) due to the nonincorporation of the drug to the nanoparticles. NP-ZN-MSZ had sizes that were major determinants of the ability of particles to passively reach inflamed intestinal mucosa and achieve maximum tissue retention times due to a greater surface area as a result of greater reactivity [5,34]. The polydispersity index that provided information on the homogeneity of the size distribution was 0.14 ± 0.1. The NPs presented negative charges with zeta potential values of −42.4 ± 5.31 mV (Table 1; Figure 4). Values less than 0.2 for the polydispersity and above 30 mV for the zeta potential (in the module) indicated a good colloidal stability in the solution, preventing the aggregation of NPs [35]. These data corroborated the findings of [10,14] that synthesized zein nanoparticles (ZNP) to encapsulate quercetin. The encapsulation efficiency (EE%) was 45%; [36] synthesized the NP-ZN using the phase separation method to encapsulate beta carotene, and obtained 55% EE.

An inflamed colon produces positively charged proteins (e.g., transferrin, eosinophilic cationic protein) [36,37,38,39]. The positive charges accumulated on the surface of the damaged epithelium offer a target for drug delivery vehicles with a negative surface charge, such as NP-ZN-MSZ. Negatively charged nanoparticles selectively target inflamed sites in the gut and gradually release the drug in response to the inflamed gut microenvironment through their electrostatic attractions [39]. The polymeric nanoparticles are biodegradable and nontoxic, and there is no organic solvent residue generated during the production process, as the 70% ethanol used for the synthesis is evaporated in the spray dryer [8].

### 3.2. Scanning Electron Microscopy (SEM)

**Figure 5:**
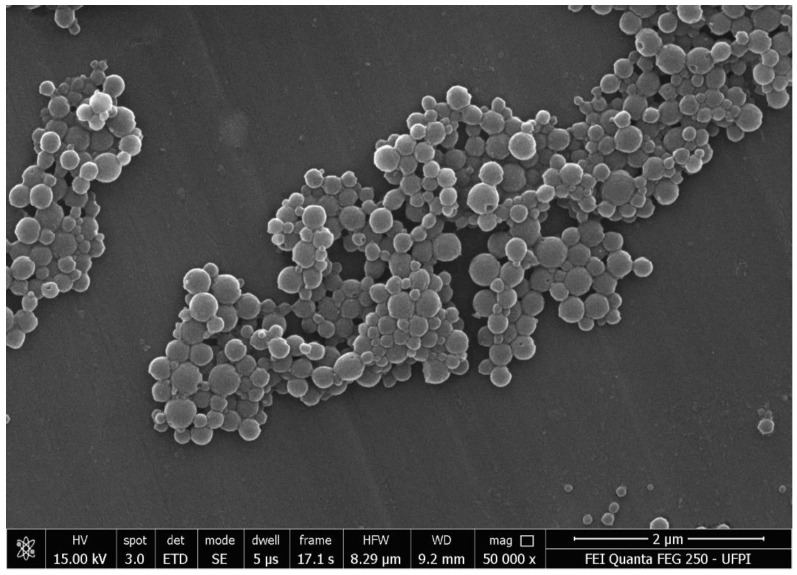
Scanning electron microscopy (SEM) of NP-ZN-MSZ.

Figure 5 shows the morphology and shape of the zein nanoparticles loaded with mesalazine, where homogeneous populations of spherical nanoparticles with a smooth surface and a size variation from 240 to 400 nm were observed, apparently similar to that calculated using DLS (Table 1). Penalva et al. (2017) synthesized zein nanoparticles as nanocarriers to improve the oral bioavailability of quercetin and demonstrated an average size of 300 nm [14].

### 3.3. Fourier Transform Infrared Spectroscopy (FTIR)

**Figure 6:**
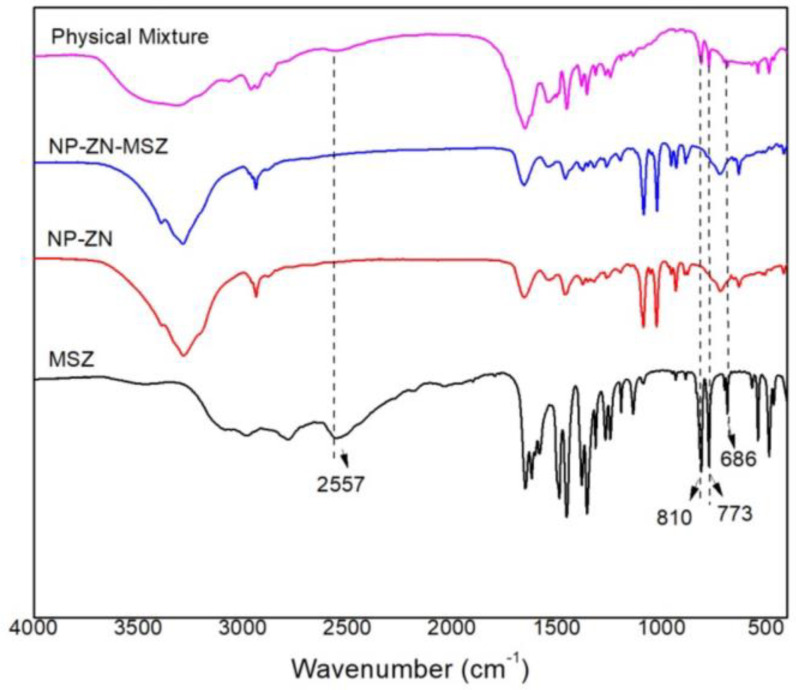
FTIR spectrum (KBr pellet) of the physical mixture of zein nanoparticles with and without the drug.

In the infrared spectrum of mesalazine (MSZ), the presence of bands that identify its functional groups was observed: 3471 cm^−1^ (N-H stretching), 2557 cm^−1^ (NH2 deformation), 1620–1590 cm^−1^ (N-H primary deformation and C-N stretch), 1246 cm^−1^ (C-N stretch), 3085 and 2978 cm^−1^ (C-H aromatic stretch), 2784 cm^−1^ (carboxylic acid OH stretch), 1647 cm^−1^ (C=O stretch of carboxylic acid), 1192 cm^−1^ (C-O stretch phenol), 810, 773 and 686 cm^−1^ (out-of-plane deformation of C-H in aromatic rings). These data corroborated the literature [40,41,42,43,44]. NP-ZN bands were observed at 3381 cm^−1^ (N-H stretch), 2935 cm^−1^ (C-H stretch), 1660 cm^−1^ (C=O stretch), 1530 cm^−1^ (N-H stretch) and 1259 (C-N stretch) [45,46,47,48]. In the physical mixture, an N-H deformation band was observed at 2557 cm^−1^, indicative of the amino group of mesalazine [44], and bands at 810, 773 and 686 cm^−1^, referring to out-of-plane C-H strains also belonging to mesalazine [40]; these bands appeared at low intensity due to the low drug:polymer ratio (1:20), and were not identified in the NP-ZN-MSZ, which showed no significant difference from the NP-ZN, indicating drug encapsulation by the nucleus prolamin without structural changes and impurities (Figure 6).

### 3.4. Thermogravimetric Analysis (TGA) and Differential Exploratory Calorimetry (DSC)

**Figure 7:**
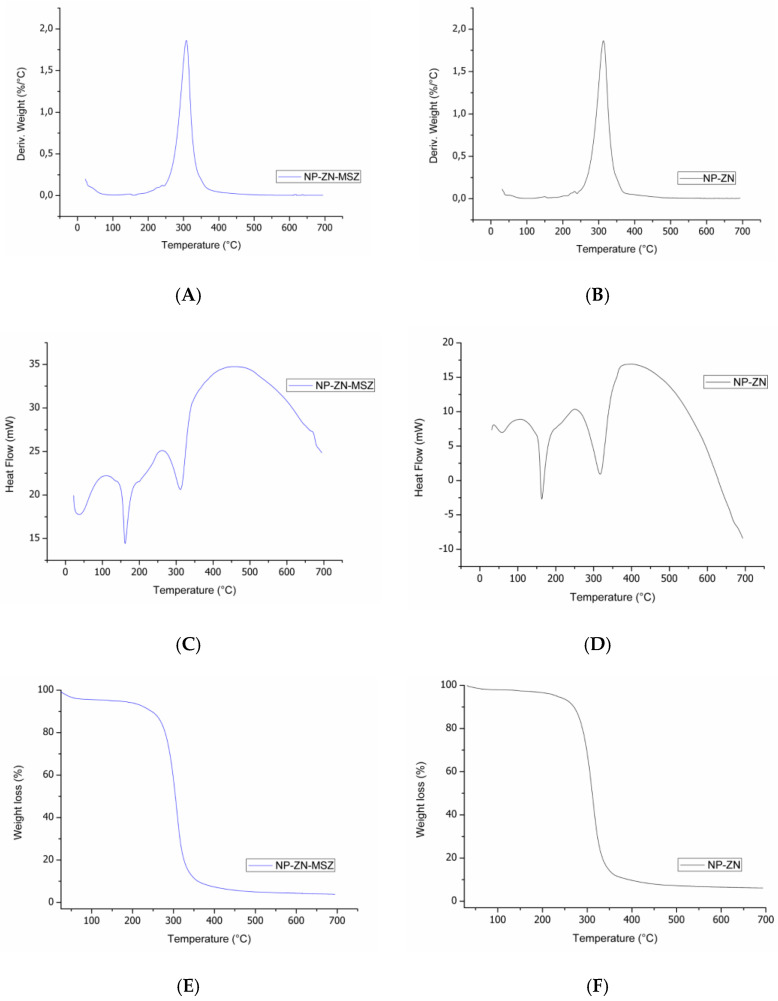
Thermogram of NP-ZN-MSZ (**A**,**C**,**E**) and NP-ZN (**B**,**D**,**F**).

The thermogram provided information on the thermal stability of the synthesized nanoparticles, which is an important aspect for in vivo drug delivery applications and for the identification of changes in the conformation of polymer chains as a function of the chemical changes undergone during the polymerization process, the synthesis of NPs or encapsulation of active ingredients [30]. In this test, the thermal stability was observed, as there was only a loss of mass from 250 °C to 350 °C (Figure 7A,B,E,F), which was consistent with the data of [45] that showed that nanocarriers could remain thermally stable at a human body temperature (37 °C) [49,50].

Differential scanning calorimetry (DSC) is commonly used to study the physicochemical state and possible interactions of the drug load in nanosystems. DSC is capable of detecting phase transitions, such as the glass transition, crystallization and melting. Endothermic melting peaks were observed [50] at 170 °C and 320 °C (Figure 7C,D) [11]. The incorporation of the drug maintained the thermal stability of the polymer, providing a satisfactory polymer–drug interaction.

### 3.5. Study of the In Vitro Release Profile

**Figure 8:**
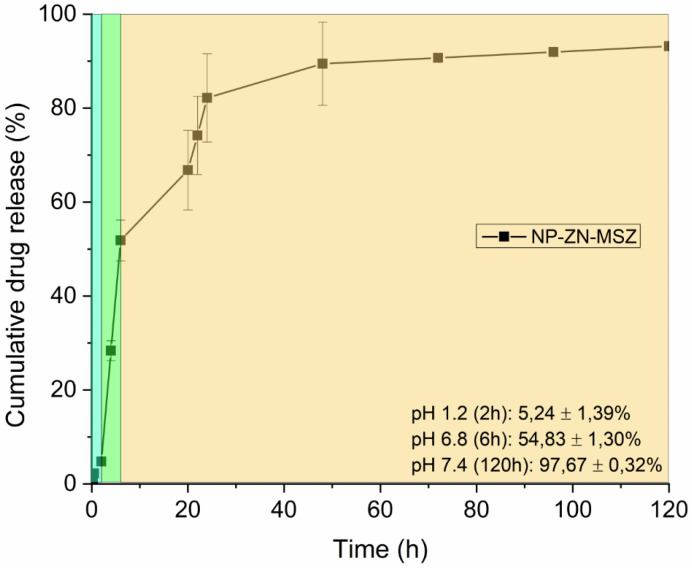
MSZ release profile of NP-ZN at pH 1.2, pH 6.8 and pH 7.4.

In vitro release studies were performed at pH 1.2, pH 6.8 and pH 7.4 at 37 °C to simulate physiological conditions when administered orally (Figure 8). At pH 1.2, in the first 2 h of the test, there was a slight release of MSZ from the nanoparticles (5.24 ± 1.39%), which suggested gastroresistance. The change to pH 6.8 during 4 h increased the release profile to 54.83 ± 1.30%, remaining at pH 7.4, where a sustained release of 97.67 ± 0.32% was observed.

In [16], the authors also observed a profile of a slow and sustained release of beta carotene from zein nanoparticles for four days (96 h); [51] also observed a sustained release profile for eight days (480 h) by encapsulating hyperoside in NP-ZN. This release profile of NP-ZN was interesting, as it may allow the drug to reach the colon rather than being released in the upper gastrointestinal tract [52].

### 3.6. Kinetics and Mechanism of In Vitro Drug Release

The study of release kinetics allowed us to understand how the drug was released from the polymer matrix [53]. For each medium, data were applied to zero-order, first-order, Higuchi, Korsmeyer–Peppas and Peppas–Sahlin mathematical models. For pH 1.2 and pH 6.8 media, the Peppas–Sahlin model (Equation (6)) showed the highest correlation coefficient (R^2^adj), and for the pH 7.4 medium, the Korsmeyer–Peppas model (Equation (7)) was the best fit (Table 2) [54].

F = K_1_t^0.5^ + K_2_t (6)

K_1_: the constant representing the contribution of the Fickian diffusion.

K_2_: the constant representing the contribution of the polymeric relaxation.

F = K_KP_·t*^n^* (7)

K: kinetic constant.

*n*: release exponent.

The Peppas–Sahlin model assumes that it is possible to calculate the diffusion mechanisms and the relaxation mechanism, as seen in Equation (6) [55]. At pH 1.2, it was observed that K_1_ (1.62) was greater than K_2_ (0.36), suggesting a contribution of the Fickian diffusion in the release. At pH 6.8, the negative K_1_ value (−5.72) and the positive K_2_ value (6.12) indicated that the release through the Fickian diffusion was inhibited, and the release mechanism was contributed through polymeric relaxation [56]. The Korsmeyer–Peppas model was used to analyze the release of polymeric dosage forms when the mechanism was not well known or when more than one type of release may have been involved. At pH 7.4, the value of n was −0.79, and for the kinetics of the drug release from swellable polymeric systems with spherical morphology (Figure 5), a value of the release exponent *n* ≤ 0.43 indicated that the release mechanism was the diffusion of the drug through the layers of the matrix, also known as the Fickian release mechanism, or “Case I” [57].

### 3.7. In Vitro Cytotoxicity Study

**Figure 9:**
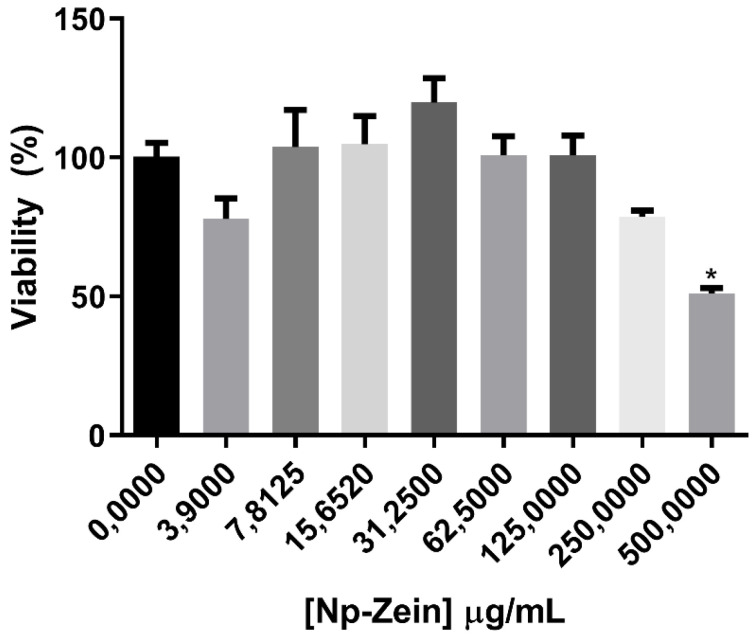
MTT assay in CT26 cells with 72 h of exposure.

Figure 9 shows the in vitro cytotoxicity study of the NP-ZN using the previously cultured CT26 (murine colon adenoma) cell line. Nanoparticles at different concentrations were added to the cell suspension and left for 72 h at 37 °C. Cell viability was measured using the MTT assay, where the nanoparticles did not show a basal cytotoxicity up to a concentration of 500 μg/mL; the usual concentration of zein used for the synthesis of NPs is 296 µg/mL, so we verified the biocompatibility of NPs.

Similar findings were obtained by [9], who used zein-based composite nanoparticles for pterostilbene encapsulation and evaluated cell viability in Caco-2, L-02 and HK-2 cells.

### 3.8. Stability Test

The colloidal stability of the NPs was monitored for 120 days after synthesis at room temperature (Table 3). No significant difference was observed in terms of the size, IPD and zeta potential, which confirmed the stability of the material and indicated the presence of particles with a lower tendency to form aggregates, an important parameter for interactions with, and the penetration of, biological barriers.

## 4. Conclusions

In this study, mesalazine was successfully incorporated into zein nanoparticles using the desolvation method, demonstrating colloidal stability. A SEM image analysis confirmed the size of the NP (220 to 400 nm). The high levels of cell viability indicated that the nanoparticles offered good biocompatibility in CT26 colonic cells. The in vitro release profile showed the gastroresistance of the nanoparticles and sustained release in the simulated ileocolonic pH 7.4 medium for 120 h. We suggest that NP-ZN, a biocompatible and biodegradable natural protein polymer, showed promising properties for the delivery of mesalazine (5-ASA) to the colon to optimize the treatment of inflammatory bowel diseases; however, further in vivo studies are needed.

## Figures and Tables

**Table 1:** Characterization of zein nanoparticles with and without the drug.

Formulation	NP-ZN	NP-ZN-MSZ
Size	218 ± 23 nm	266.6 ± 52 nm
Polydispersity index	0.20 ± 0.1	0.14 ± 0.1
ζ Potential	−39.3 ± 2 mV	−42.4 ± 5.31 mV
Drug loaded	-	43.8 µg/mg de NP
Encapsulation efficiency	-	45%
Yield	70%	65%

**Table 2:** Representation of experimental data from the kinetic models that best fit the different release media.

	Peppas–Sahlin Model		
Medium	R^2^adj	K_1_	K_2_
pH 1.2	0.99	1.62	0.36
pH 6.8	0.92	−5.72	6.12
	**Korsmeyer–Peppas Model**		
**Medium**	**R^2^adj**	**K**	***n***
pH 7.4	0.82	99.3	−0.79

**Table 3:** Colloidal stability of NP-ZN-MSZ after 120 days at room temperature.

Time (Days)	Size (nm)	Polydispersion Index	Zeta Potential (mV)
0	266.6 ± 52 nm	0.14 ± 0.1	−42.4 ± 5.31 mV
30	296.2 ± 25 nm	0.11 ± 0.02	−40.4 ± 6 mV
60	300.6 ± 81 nm	0.12 ± 0.1	−41.6 ± 9.3 mV
90	269 ± 31 nm	0.14 ± 0.1	−37.4 ± 8.24 mV
120	291.6 ± 32 nm	0.13 ± 0.03	−32.4 ± 6.7 mV

## Data Availability

Not applicable.
